# Case report: Successful treatment of hyperbaric oxygen for radiation-induced hemorrhagic cystitis in a 95-year-old patient with bladder cancer

**DOI:** 10.3389/fonc.2025.1410148

**Published:** 2025-02-03

**Authors:** Li Lin, Man He, Yanyan Zeng, Xiaoxiao Ni, Yequn Guo, Xiaojuan Xie, Lingling Sun, Huai Huang

**Affiliations:** ^1^ Guangzhou University of Chinese Medicine, Guangzhou, China; ^2^ Department of Hyperbaric Oxygen Medicine and Rehabilitation, General Hospital of Southern Theater Command of PLA, Guangzhou, China

**Keywords:** hyperbaric oxygen therapy (HBOT), bladder cancer, radiation-induced hemorrhagic radiation cystitis, late radiation cystitis, hematuria, elderly patient

## Abstract

**Background:**

Severe radiation-induced hemorrhagic cystitis (RHC) may ultimately require radical cystectomy. High-quality clinical evidence exists regarding the impact of hyperbaric oxygen therapy (HBOT) on RHC. However, there is a lack of reported data addressing the applicability of HBOT in very elderly patients (over 90 years old) with severe bleeding necessitating multiple blood transfusions.

**Case presentation:**

The patient is a 95-year-old male who suffered from severe hematuria due to RHC after 33 sessions of radiotherapy for muscle-invasive bladder cancer (T2N0M0, stage: II). After a series of subsequent therapies that failed to control the hematuria, the patient received 196 HBOTs between 11 October 2022 and 23 October 2023. Following a progressively adjusted HBOT protocol, the patient’s hematuria (urine red blood cell (RBC) count showed 210,859 RBC/μL at maximum) was effectively controlled, and his symptoms subsequently resolved after 69 HBOTs. After the 196th HBOT, pelvic MRI revealed a reduction in the size of the lesion on the left posterior wall of the bladder compared to prior assessments. This reduction was suggestive of diminished intravesical hematoma. As of 26 October 2023, it is noteworthy that the patient’s hemoglobin level has restored to the pre-hematuria level (106 g/L) with no need for blood transfusions for 8 consecutive months. Moreover, there was no recurrence of hematuria during the follow-up period. To the best of our knowledge, this report represents the first documented case of hematuria resolution in a patient over 90 years old with severe RHC.

**Conclusion:**

This case represents the first documented instance of successful hematuria resolution in a patient over 90 years of age with severe RHC. The positive therapeutic outcome achieved with personal protocol (1.4-1.6 ATA) in this 95-year-old patient, expands hyperbaric oxygen pressure options for elderly patients. Moreover, it also records a comprehensive dataset, including the time of the patient’s recurrence of hematuria and the subsequent trend of hematuria control, which contributes valuable evidence to the research on HBOT for the repair of bladder mucosal tissue.

## 1 Introduction

Bladder cancer is the 9th most commonly diagnosed cancer worldwide, with approximately 614,298 new cases and 220,596 deaths in 2022 (1). Based on the spread and depth of bladder wall invasion of the tumor, bladder cancer is classified into non-muscle-invasive bladder cancer (NMIBC) and muscle-invasive bladder cancer (MIBC), which approximately account for 75% and 25% of cases, respectively (2). The standard treatment for the former is transurethral resection of bladder tumors (TURBT) followed by intravesical chemotherapy or immunotherapy. For the latter, because the tumor is highly invasive and has penetrated deeply into the bladder wall, radical cystectomy and pelvic lymph node dissection is the current gold standard in eligible patients. For the patients who are unable to undergo radical cystectomy due to physical or psychological factors or who have a strong desire to preserve their bladder, the most effective treatment currently is a three-in-one combination of TURBT, radiation therapy, and chemotherapy (3). Radiation therapy, to kill cancer cells, is often a primary treatment when surgery isn’t a selection or isn’t desired. Although radiation therapy can achieve good disease control and maintain bladder function, it is likely to lead to the occurrence of radiation cystitis, presenting frequent urination, nocturia, and even hematuria – a severe symptom could also result in radical cystectomy. Radiation cystitis is categorized into acute radiation cystitis and late radiation cystitis (LRC) based on the timing of onset (4). Acute radiation cystitis typically manifests within weeks or months following radiotherapy. Its symptoms commonly include frequency, urgency, dysuria, hematuria and pain. In most cases, the symptoms are mild severity, self-limiting, and progressively improved after the completion or cessation of radiotherapy through conservative treatment. LRC manifests months to years after radiotherapy and is characterized as a chronic and progressive ailment. The symptoms encompass hematuria, frequency, urgency, urinary incontinence and dysuria, among which hematuria is the predominant symptom. The severity of these conditions varies from mild to severe, and in certain cases, these conditions can pose life-threatening risks. Despite its relative rarity, the incidence of LRC ranges from 5 to 15% (5) and LRC can lead to severe and challenging-to-treat hemorrhages, further leading to life-threatening hypovolemic shock (6). In some studies, severe radiation-induced hemorrhagic cystitis (RHC) has been associated with a 44% mortality rate despite aggressive treatment (7).

Hyperbaric oxygen therapy (HBOT) is a treatment procedure that involves breathing 100% O_2_ for a certain time under a certain pressure (8). Mainous et al. (9) pioneered the application of HBOT in the treatment of tissue damage resulting from radiotherapy, suggesting that HBOT can enhance the healing of damaged tissues after radiotherapy. Weiss et al. (10) subsequently reported the initial use of HBOT in three patients with severe symptoms of RHC. The reported success rate of HBOT for RHC varies from 60% to 92% (11). When initiating HBOT implemented within 6 months of hematuria onset, the likelihood of complete or partial symptomatic improvement is up to 96% (12). HBOT mobilizes stem cells to enhance neovascularization, also can facilitate tissue healing, and prevent complications following surgery and radiotherapy (5, 13, 14). Presently, HBOT is considered the treatment capable of reversing macroscopic changes in the urinary bladder induced by radiotherapy (15). Evidence-based medical research has also indicated that HBOT promotes recovery from radiation damage to bones and soft tissues, offering accurate efficacy with minimal complications. Given the severity of bladder wall damage, HBOT is a treatment option for severe patients with RHC (16). Based on the established clinical efficacy of HBOT in the management of RHC, both the European Committee on Hyperbaric Medicine (17) and the Hyperbaric Oxygen Medicine Branch of the Chinese Medical Association (18) have officially recommended HBOT for the treatment of RHC. Furthermore, HBOT was incorporated into the “Guidelines for the Diagnosis, Prevention, and Management of Chemical Cystitis and Radiation Cystitis” by the British Association of Urological Surgeons (19). In the context of domestic recommendations, the China Radiotherapy Oncology Alliance released the “Clinical Practice Guidelines for the Prevention and Treatment of Radiation Bladder Injury”, categorizing HBOT as a strong recommendation with high-quality evidence for late radiation-induced bladder injuries (20).

Recently, Oscarsson et al. (15) designed a pivotal randomized controlled trial to assess the efficacy of HBOT on LRC. Their high-quality clinical evidences suggested that HBOT, a safe and well-tolerated therapy, could relieve symptoms of LRC. Notably, the trial included patients with ages up to a maximum age of 80 years and excluded patients with severe bleeding who required multiple transfusions. In the current article, we present a successful application of HBOT in curing a 95-year-old patient with severe RHC after radiation therapy for MIBC (T2N0M0, stage: II).

## 2 Case presentation

### 2.1 Patient information

**Figure 1:**
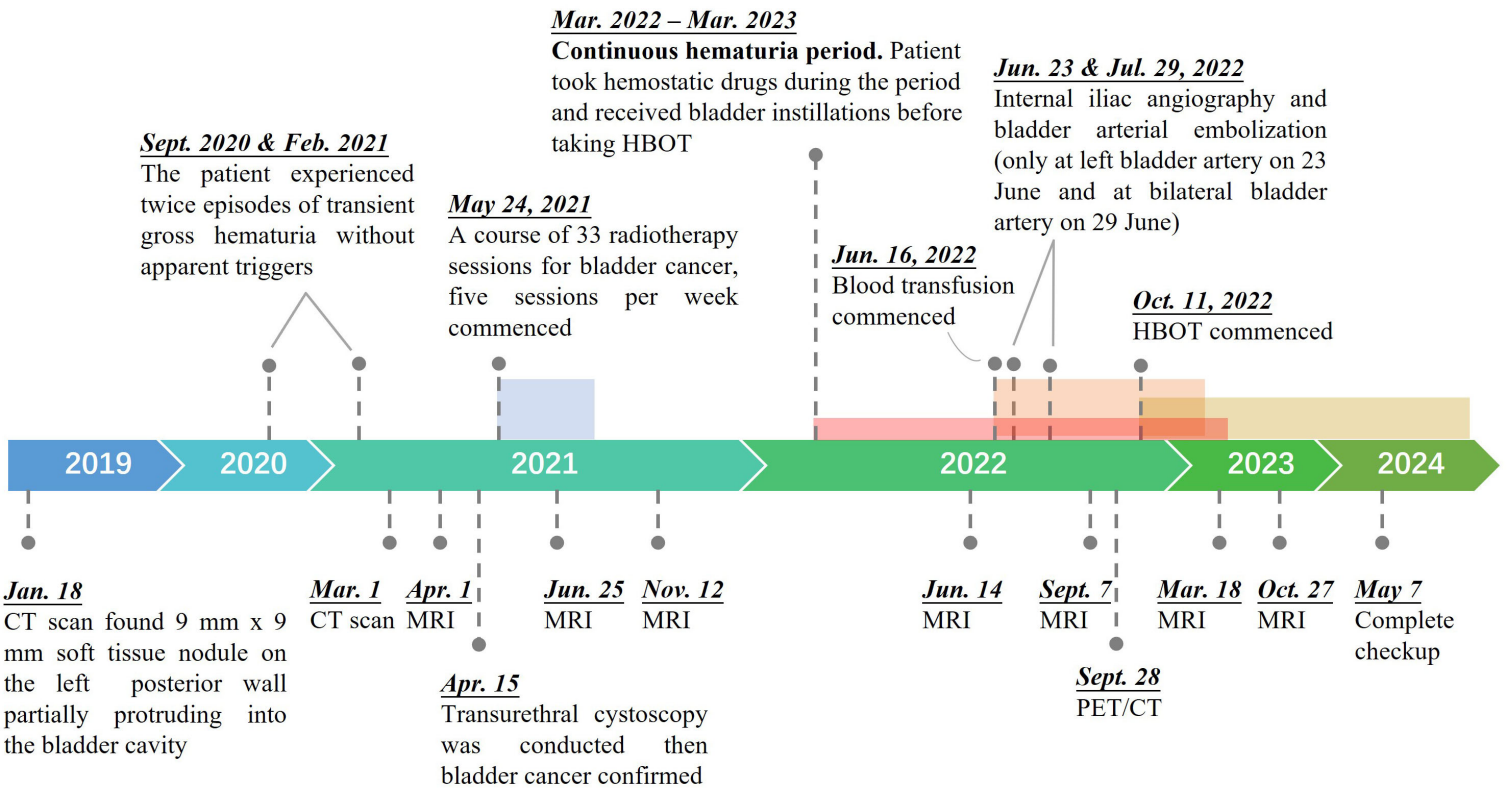
Treatment timeline of the 95-year-old patient from onset to recovery of bladder cancer. A color zone denotes a period of a specific treatment, which is presented by the pin in front of the zone.

The patient is a 95-year-old male. As shown in **Figure 1**, a pelvic CT examination revealed the presence of new lesions on the patient's left posterior wall of the bladder as early as January 2019. The examination revealed slight thickening of the posterior wall of the bladder, with an approximately 9×9 mm soft tissue nodule on the left posterior wall partially protruding into the bladder cavity. In both September 2020 and February 2021, the patient experienced episodes of transient gross hematuria without apparent triggers. On 1 March 2021, the re-examination results of the pelvic CT suggested a diagnosis of bladder cancer. Subsequently, transurethral cystoscopy was conducted for further diagnostic evaluation and treatment on 15 April 2021. Pathology of tissue removed from the bladder mass revealed that the tumor on the left wall of the bladder was consistent with low-grade papillary urothelial carcinoma (T2N0M0, stage: II). Considering huge potential risks of surgery and the patient’s strong desire for bladder preservation, we performed the patient a course of 33 radiotherapy sessions for bladder cancer, administered five sessions a week, commencing on 24 May 2021. Compared with pelvic MRI data obtained on 1 April 2021, the pelvic MRI data obtained on 25 June 2021 showed significant changes after 5 weeks of radiotherapy, as the space occupied by the left posterior wall of the bladder had shrunk. However, in March 2022, approximately 10 months after radiotherapy, the patient developed gross hematuria and was diagnosed with LRC. To control his hematuria, the patient has taken hemostatic drugs (Yunnan Baiyao), intravesical instillation (alternating use of sodium hyaluronate and nitrofurazone), moreover, the patient underwent internal iliac angiography and bladder arterial embolization twice (one was on 23 June 2022 on the left bladder artery and the other was on 29 June 2022 on the bilateral bladder artery). The patient also underwent multiple transfusions due to the anemia caused by blood loss since 16 June 2022. However, his hematuria soon recurred and got even worse, as the two operations only brought about the disappearance of hematuria for 1 week and 3 months, respectively. The pelvic MRI on 7 September 2022 (**Supplementary Figure S1**) showed that the left upper wall of the bladder had diffused T2WI+FS signal enhancement and that the wall was slightly thickened, indicating LRC and hemorrhage. The patient started to receive HBOT on 11 October 2022 after all other treatments for hematuria were ineffective excluding cystoscopy fulguration due to the high risk of bladder perforation.

### 2.2 HBOT protocol

**Figure 2:**
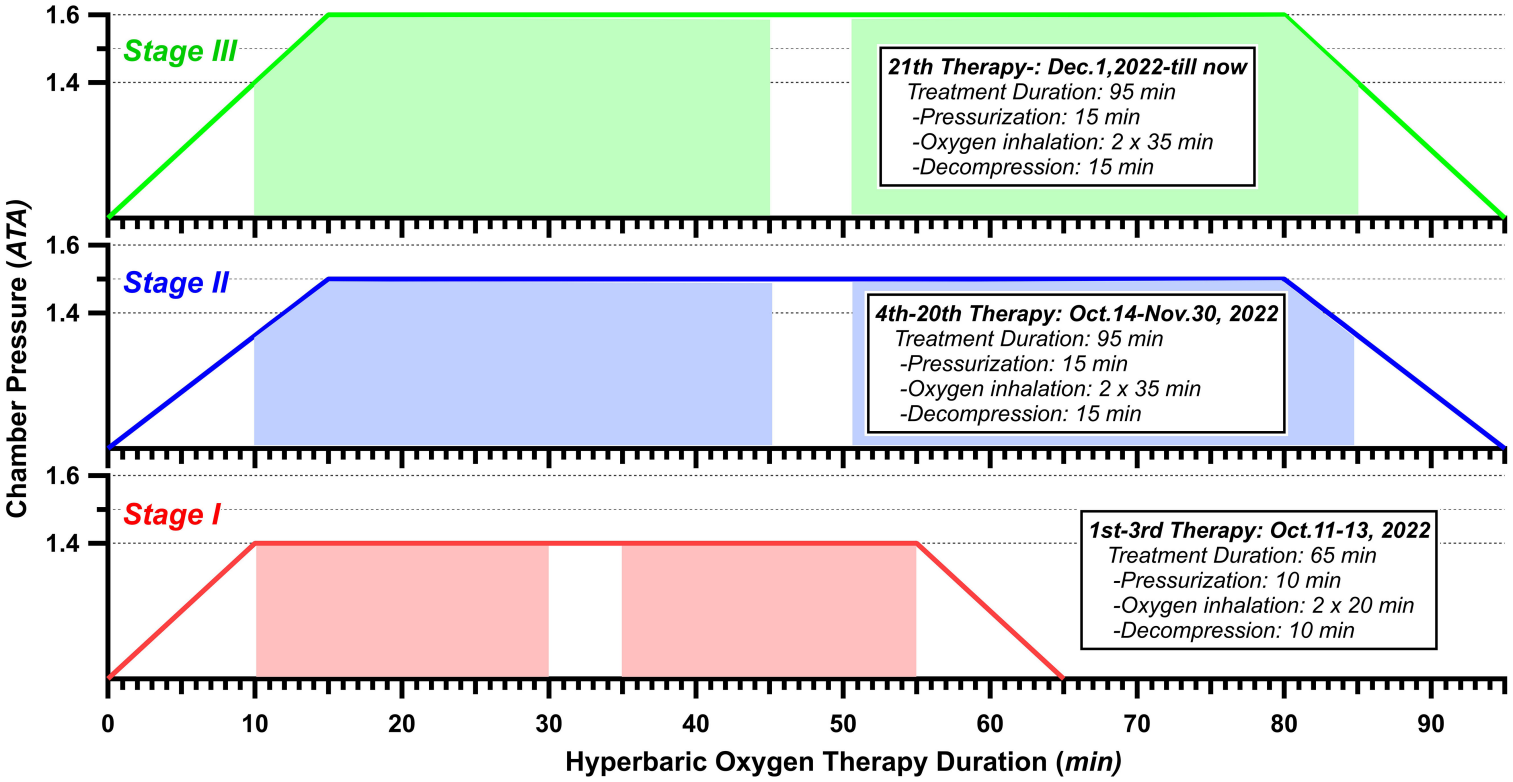
Hyperbaric oxygen therapy protocol. Color-overlay timeline represents the time of treatment.

HBOT typically involves pressures exceeding 1.4 ATA, with common treatment ranges between 2.0 and 2.5 ATA, administered for 90 to 120 minutes per session (21). However, there is no standardized treatment protocol, and the specific parameters may vary across studies depending on the patient’s condition and symptom progression. Treatment intensity is often adjusted based on individual patient tolerance and therapeutic response. According to the existing reports (22, 23), considering the patient’s age, the severity of symptoms, and the opinions of the family, we carefully worked out a HBOT protocol (**Figure 2**). From 11 October 2022 to 23 October 2023, HBOT was implemented a total of 196 sessions. The HBOT protocol involved three distinct stages. StageI (Adaptation Period): During the initial 3 treatments, the patient underwent HBOT with 100% oxygen at 1.4 atmospheres absolute (ATA) for a short duration of 65 minutes each; Stage II (Initial Period): For treatments 4 through 20, a protocol was followed consisting of 100% oxygen at 1.5 ATA for a duration of 95 minutes each; and Stage III (Conventional Period): Treatments 21 through 196 followed by 100% oxygen at 1.6 ATA for a duration of 95 minutes each. In each stage, there was a 5-minute air break during the treatment. In Stages I, the patient inhaled oxygen for 2×20 minutes under steady pressure (1.4 ATA). In Stage II/III, apart from the pressure variance, the patient commenced oxygen inhalation 10 minutes after the treatment initiation, while the chamber was still undergoing pressurization. Similarly, the patient also maintained oxygen inhalation until 5 minutes after decompression had begun. Therefore, the length of oxygen inhalation was 2×35 minutes in each treatment of Stage II/III.

## 3 Results

**Figure 3:**
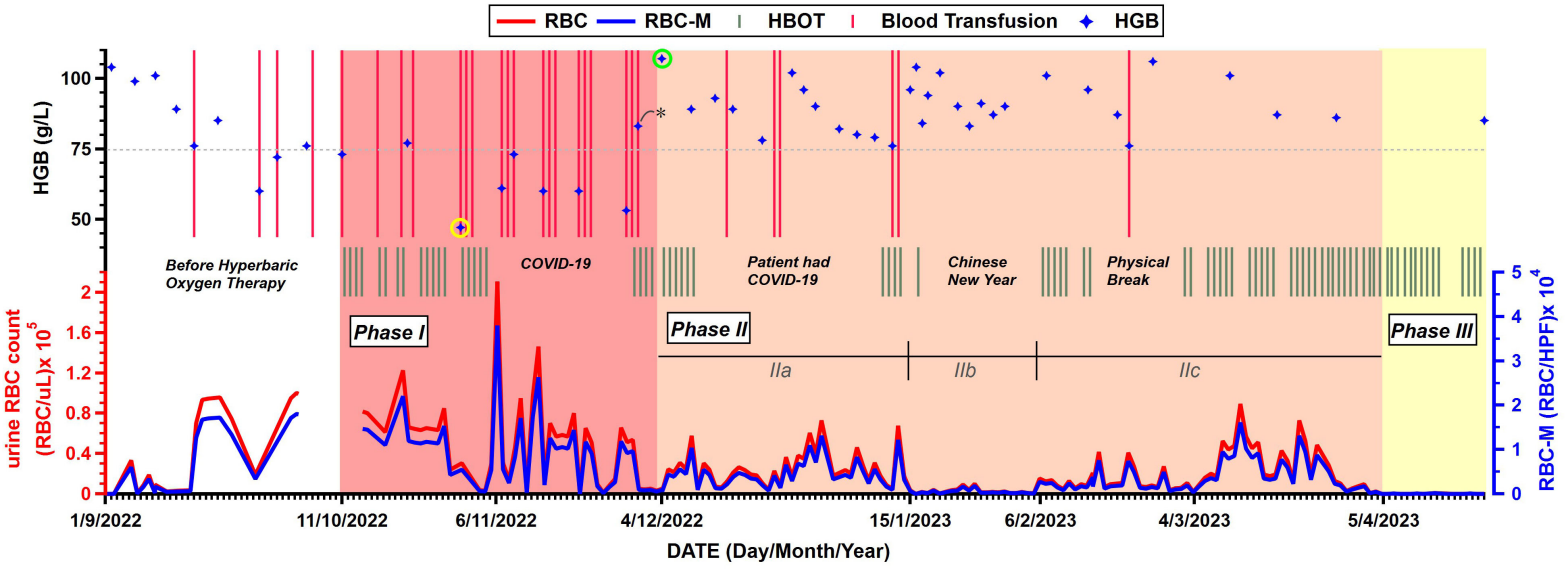
Changes in relevant indicators before and after hyperbaric oxygen therapy (HBOT). Parameters include timelines of patient’s blood transfusion and hemoglobin (HGB) levels from routine bloodwork (Top chart), HBOT (Middle chart), urine red blood cell (RBC) count and urine RBC count examined under a microscope (RBC-M) (Bottom chart). RBC/μL, red blood cells per microliter; RBC/HPF, red blood cells per high power field. Stars with yellow cycle and green cycle denote the lowest and highest HGB levels, respectively. The star with asterisk denotes the only HGB value measured after blood transfusion. (All data of the figure is available from **Supplementary Tables S1~S4** in **Supplementary Materials**).

Before receiving the first HBOT on 11 October 2022, the patient had developed gross hematuria for more than half a year and had received 12 sessions of blood transfusion due to the anemia (**Supplementary Table S1**). As shown in **Figure 3**, the period during which the patient received HBOT was classified into three phases.

### 3.1 Phase I

The most serious period, presented by red color zone in **Figure 3**, had lasted nearly 2 months from October 11 to December 3, 2022. In Phase I, the mean urine RBC count was high to 49,415 RBC/μL as well as 8,895 RBC per high power field (RBC/HPF) when the urine was examined under a microscope. On 31 October 2022 (the 14th HBOT day), routine bloodwork reported an extreme low hemoglobin (HGB) level at 47 g/L (presented by a star with yellow cycle in **Figure 3**), which triggered a thrice-weekly blood transfusion protocol to compensate for the blood loss. The patient’s HBOT treatment was interrupted by COVID-19 after 18 HBOTs and two days later, the patient’s hematuria was recorded at the maximum count (RBC:210,859 RBC/μL, RBC-M:37,954.6 RBC/HPF) during the entire treatment period. Meanwhile, the patient’s HGB levels were rarely higher than 75 g/L in Phase I.

### 3.2 Phase II

From 4 December 2022 (the 23rd HBOT day) to 4 April 2023, presented by pink color zone as Phase II in **Figure 3**, the patient’s hematuria improved significantly compared to that in Phase I. On 4 December 2022, the patient’s HGB level was 107 g/L (presented by a star with green cycle in **Figure 3**), which was recorded to be above 100 g/L for the first time since the initiation of HBOT. Compared to 83 g/L four days earlier (the only value measured after blood transfusion which was represented by a star with asterisk in **Figure 3**), the improvement is intuitively attributed to a 5-day of non-severe hematuria period, which may assume to the effects of the HBOT become apparent. During the period, three interruption periods hampered continuity of HBOT treatment, including the period during which the patient himself was infected with COVID-19. The mean urine RBC count in Phase II was 18,950 RBC/μL and 3,411 RBC/HPF and decreased 62% compared to Phase I, which means a significantly improvement for the patient. Moreover, the patient’s HGB levels were all above 75 g/L which high-frequency blood transfusions were not needed anymore. Phase II had lasted 4 months and was re-classified into 3 sub-phases (IIa, IIb and IIc) based on the severity of the patient’s hematuria.

#### 3.2.1 Phase IIa

It had lasted 42 days, the patient’s hematuria continued the trend of Phase I and his urine RBC count remained at a high level. During this period, the patient received 5 more blood transfusions and 10 HBOTs, and the mean urine RBC count was 24,421 RBC/uL and 4,396 RBC/HPF, decreased 50% compared to Phase I.

#### 3.2.2 Phase IIb

Starting from 15 January 2023, the patient’s urine RBC count decreased from ten-thousands per microliter level to thousands per microliter level. As it happened to be the Chinese New Year period, the patient only received one HBOT as it had lasted 22 days.

#### 3.2.3 Phase IIc

However, the patient’s hematuria worsened again on 6 February 2023 with the urine RBC count increasing from 648 RBC/uL to 10,591 RBC/uL. The patient immediately continued to receive the HBOT. After nearly two months and 35 HBOTs, the patient’s urine RBC count dropped to dozens per microliter level, following a “low-high-low” trend.

### 3.3 Phase III

Since 5 April 2023, the patient’s hematuria had finally disappeared after receiving 69 HBOTs, as his routine urinalysis showed that the RBC counts were only in the hundreds per microliter and continuously decreased to dozens per microliter then have remained so till now without recurrence any more. This period was defined as Phase III (presented by yellow color zone in **Figure 3**). By the time the patient was discharged from the hospital on 2 June 2023, the cumulative number of HBOTs was 120 sessions. His HGB level has restored to the pre-hematuria level (106 g/L), and his urine RBC count has also kept at the dozens per microliter. In the following days, the patient continued HBOTs five sessions per week, accompanied by routine bloodwork and routine urinalysis. To date, he hasn’t needed blood transfusions for 8 consecutive months. The routine bloodwork on 26 October 2023 showed the HGB level of 106 g/L. Besides, there were no treatment-related complications or adverse effects observed.

**Figure 4:**
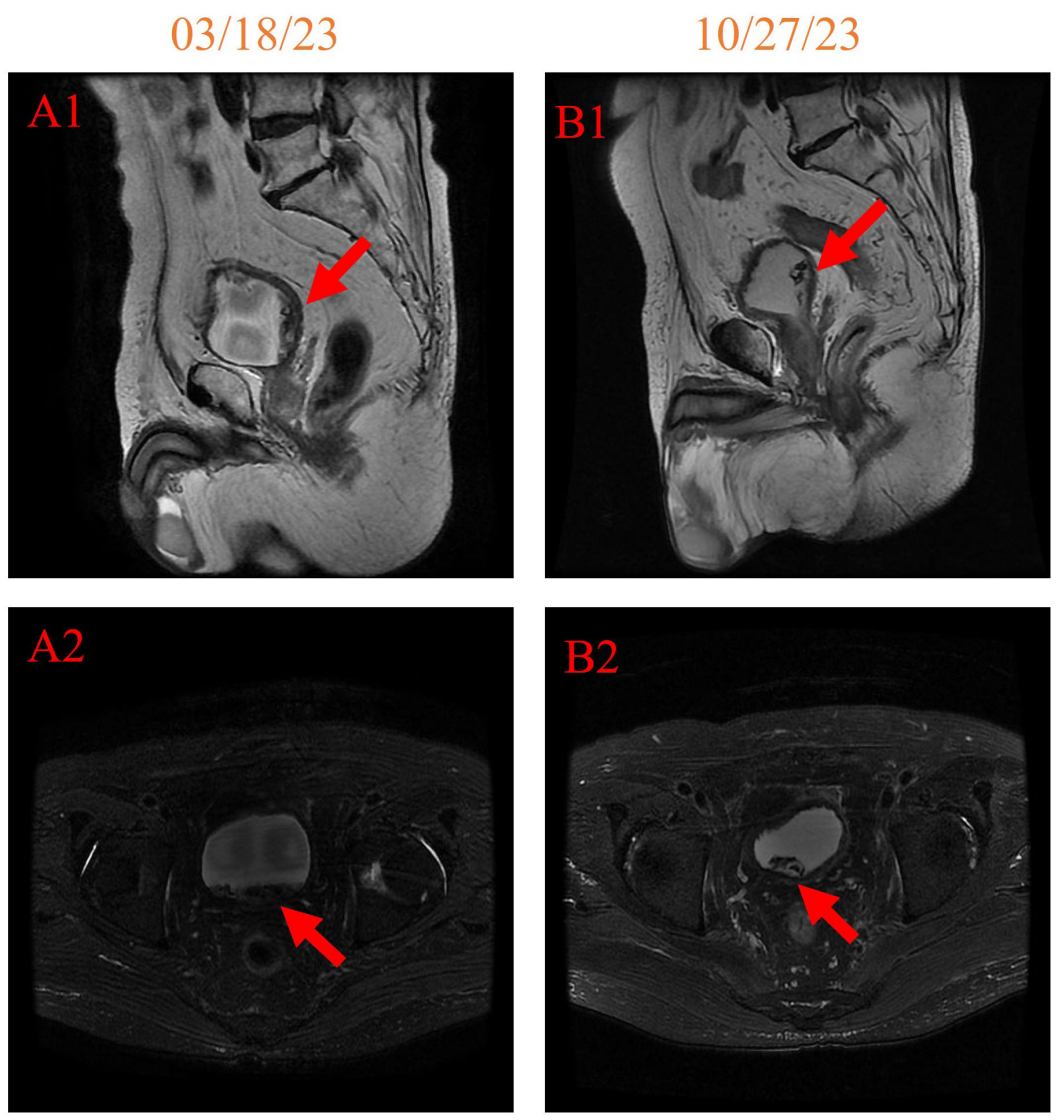
pelvic MRI imaging on 18 March 2023 [**(A1, A2)** 158 days after initiating HBOT] and 27 October 2023 [**(B1, B2)** 381 days after initiating HBOT].

In addition, notable improvements were observed when comparing the pelvic MRI on 27 October 2023 with that on 18 March 2023 (**Figure 4**). The space occupied by the left posterior wall of the bladder shrank, and the hemorrhage in the bladder cavity has also reduced. These findings collectively indicate positive progress in the patient’s pelvic region.

## 4 Discussion

The patient was diagnosed with bladder cancer then received 33 sessions of radiotherapy. Ten months later, the patient developed persistent severe hematuria, which was diagnosed with LRC. The pathophysiology of LRC is not fully understood (11). Previous studies (4, 11, 24) reported that the density of blood vessels and cells in the bladder urothelium and smooth muscle layer decreases due to radiotherapy. Damage to endothelial cells and the presence of perivascular fibrosis led to ischemia and obliterative arteritis obliterans and consequently to the formation of bleeding ulcers. Chronic inflammation and fibrosis are characteristic features of LRC. These conditions will impair the complex neuromuscular interactions responsible for proper urine storage and release, resulting in symptoms related to voiding. Poor tissue oxygenation and tissue ischemia contribute to the development of necrotic and sloughed tissue, accompanied by fibroblast deposition in the ischemic tissue layer. Simultaneously, compensatory neovascularization and telangiectasia lead to the formation of a fragile vascular network that is prone to rupture as the bladder contracts, potentially causing refractory hematuria. Till now, the treatment of LRC is challenging. Its initial management typically involves a multifaceted approach, including hemostatic drugs, the administration of oral or intravenous fluids, blood transfusion if indicated, transurethral catheterization with bladder washout and irrigation, etc. In the case of severe hematuria, cystoscopy fulguration or intravesical instillation may be employed to control the bleeding. If initial interventions prove ineffective, surgery, such as embolization therapy, may be pursued (11). Although such methods can have short-term effects, they have a high recurrence rate and are likely to cause more serious adverse effects, such as bladder necrosis, fistulas, and fibrosis. Indeed, the patient had received hemostatic drugs (Yunnan Baiyao), intravesical instillation (alternating use of sodium hyaluronate and nitrofurazone), embolization and other hemostatic methods for half a year (**Figure 1**) except for cystoscopy fulguration due to the high risk of bladder perforation, but the effects of such methods were short-lived and could not completely cure hematuria. If the hematuria can’t be improved, the worst option that cystectomy (removal of the bladder) and urinary diversion procedures cannot be avoided.

In comparison to alternative treatments, HBOT boasts a high patient acceptance rate, ensuring safety, minimal discomfort, precise therapeutic efficacy, and fewer adverse reactions. HBOT has demonstrated effectiveness in ameliorating late radiation-related tissue damage in various tissues, and there is reliable evidence supporting its efficacy in treating RHC (16). Although HBOT was recommended to applied for RHC in the United States, Europe, and China, there is currently no standardized treatment protocols. Various clinical studies have adopted different treatment regimens, each based on the patient’s specific condition and symptom improvement. The general protocol is 1.8~3.0 ATA for 60~120 min for approximately 30 sessions (25). Particularly, for the case of the 95-year-old who had a frail constitution and organ degeneration and a history of hypertension, a personalized and dynamic HBOT protocol plays a crucial role. According to Leveque et al. (23) the therapeutic mechanisms of action at 1.4 ATA and 2.5 ATA are similar. Therefore, it can be reasonably inferred that HBOT at 1.6 ATA likely produces similar mechanisms of action. After comprehensive considerations, our team worked out a protocol containing 3 stages with lower pressures than general (see **Figure 2** and section of HBOT protocol). From initiating HBOT on 11 October 2022 until 5 April 2023, the patient’s hematuria significantly improved after 69 sessions of HBOT. As Cardinal et al. (26) performed meta-analysis on reviewing seven studies then found the average time to recur for RHC ranged from 3 to 120 months. Dellis et al. (22) reported that additional HBOT was administered after a recurrence occurred during the follow-up period. Given that this patient is extremely elderly and had severe hematuria, there is insufficient research on the exact duration needed to fully repair the bladder mucosa in such cases. In order to consolidate the treatment effect, and taking into full consideration the efficiency of HBOT in improving patient’s physiological and personal quality of life (15), no patient’s complaints on adverse effects such as oxygen poisoning under the protocol with lower pressures (27) and no financial burden (28) to the patient which was supported by relevant fundings, we agreed the patient to continue the HBOT. The patient who experienced from Phase I to Phase III with detailed records, was discharged from the hospital on 2 June 2023 when his urine RBC count had been keeping at dozens per microliter for some time. Since then, the 95-year-old male (turn to 96 in 2024) still receive HBOT, but the frequency of HBOT has gradually decreased. We conducted long-term follow-up for the patient, with regular outpatient visits for comprehensive evaluations, including symptom assessment, bladder function, imaging, treatment response, and quality of life. Additionally, the patient can communicate remotely with the healthcare team via social media or telephone. We emphasize the importance of closely monitoring symptoms such as urination and pain, as well as changes in social, emotional, and physical well-being. Family members are encouraged to provide active psychological support. This follow-up not only aids in evaluating the effectiveness of HBOT but also facilitates the early detection and management of potential complications, ensuring optimal treatment outcomes and quality of life.

The process of the patient’s hematuria was from onset to severity to relief, recurrence and finally cure via HBOT. The mechanism of action of HBOT may involve circulating plasma rich in highly saturated oxygen increasing the oxygen partial pressure of blood and tissues, which can ameliorate the hypoxic conditions of radiation-damaged tissues. Then, aerobic metabolism is enhanced, tissue edema is reduced, fibroblast proliferation is fostered, and leukocyte activity is augmented. The combined effects further stimulate the formation of granulation tissue at the injured site, promoting the repair of bladder mucosa, accelerating ulcer healing, and ultimately achieving the goal of controlling bleeding (29). HBOT clinically improves symptoms related to late radiation-related tissue damage, which typically manifests 2~3 months after HBOT is initiated (15). In this case, it took 3 months (11 October 2022~15 January 2023) for the urine RBC count to decline to a lower level (from ten thousand per microliter to thousand per microliter as shown in **Figure 2.IIb**), which is consistent with the conclusions of previous reports (15). From the results, the patient’s urine RBC count maintained at mean value of 2,694 RBC/uL and 485 RBC/HPF for a brief period (**Figure 3.IIb**) until it increased again on 6 February 2023. we named the afterwards period of recurrence period as shown in **Figure 3.IIc**. Compared to recorded recurrence interval ranging from 3 to 120 months (26), the 95-year-old patient only experienced about 3 weeks’ steady period. As for the existence of the recurrence period in Phase IIc, it may relate to the hysteresis of bladder mucosal repair. With continuing HBOTs, the above mechanism continues until the completion of ulcer healing. The patient’s urine RBC count finally declined to dozens per microliter and has remained free of recurrence so far and his gross hematuria and dysuria eventually disappeared (**Figure 3**). Besides, the absence of adverse reactions during and after HBOTs is another significant indicator to evaluate the efficacy of the therapy. Following discharge from the hospital, the patient regained his self-care ability, independent mobility, and engaged in basic exercises such as chest expansion, achieving a significant improvement in quality of life. The implementation of this therapy yielded extremely positive results on the 95-year-old patient. Not only does it show that HBOT can be an effective treatment and should be considered a reliable method for treating severe hematuria caused by RHC, but it also broadens the age range for which HBOT is applicable.

Fortunately, in this case, our patient didn’t have any complaints of adverse effects and complications during the HBOT period. In general, the adverse reactions of HBOT include barotrauma, decompression sickness, oxygen poisoning, etc., and some rare complications such as cerebral thrombosis and cerebral hemorrhage may also occur (30). To prevent the potential risks, it primarily requires strict implementation of the personal HBOT protocol including treatment duration, pressurization, decompression and intermittent air breaks. Besides, a sensitivity test on patients who are sensitive to oxygen before HBOT and some antioxidants are necessary to prevent oxygen poisoning. Minimizing activities during and after decompression and keeping the limbs relatively steady are also effective on preventing decompression sickness. Dripping 1% ephedrine into the nose and doing action like swallow, yawn, close mouth and pinch nose to blow air can keep the pressure inside and outside the tympanic cavity balanced during the pressurization process to prevent barotrauma, etc. As for the elderly patients, they are physically weak, have difficulty in moving, and some of them have hearing, speech disorders, unconsciousness and other diseases. Their poor adaptability and responsiveness to the new environment, especially in a confined space, may make their diseases worse. Therefore, it is necessary to check their conditions before receiving HBOT. A personal HBOT protocol based on the elderly patient’s anamnesis and comorbidities is significant and requires strict implementation to prevent adverse reactions. Elderly patients should also be fully respected and kindly treated, for example, explaining questions and introducing the relevant discomfort and treatment methods patiently and meeting their reasonable needs etc. For elderly patients who needs caregiver, actively cooperation from multiple parties is required to ensure the safety of treatment and avoid risks. If the patient shows symptoms such as convulsions and seizures, it must stop oxygen inhalation and change to air inhalation immediately. Reducing the oxygen pressure and chamber pressure gradually and paying close attention to the patient’s breathing condition to avoid pulmonary barotrauma due to laryngospasm or breath holding, etc. To call for the doctor for emergency treatment to ensure the patient’s life safety. The application scope of HBOT is expanding nowadays through repeated clinical practice and research. Its mechanism of action, scope of application, treatment protocols for various diseases and prevention of toxic adverse effects still need further research, discussion and improvement, so that HBOT can play an increasingly important role in the medical field.

One limitation of this case report is the lack of baseline data on the patient’s sleep, cognitive function, and quality of life assessment prior to treatment. However, we maintained regular communication with the patient and his family throughout the treatment process. According to their feedback, the patient experienced significant improvements in cognition, sleep, and overall quality of life following HBOT, compared to the period before treatment. Incorporating these assessments into the treatment outcome evaluation for future cases would be valuable. Another limitation is that this is a single case report, which limits its generalizability. Even though, the successful treatment shows that, with the participation of personalized treatment protocol, HBOT can be incorporated into the treatment regimen of elderly patients with RHC to mitigate the adverse effects of radiation-induced soft tissue damage.

## 5 Conclusion

Our study presents a successful application of HBOT in curing a 95-year-old patient with severe RHC after radiation therapy for MIBC (T2N0M0, stage: II). Following a personal HBOT protocol (1.4-1.6 ATA), the hematuria of the patient disappeared after 69 sessions of HBOT and has remained free of recurrence so far. The patient continued HBOT in follow-up period and hasn’t reported any adverse effects and complications. As a safe and well-tolerated therapy, HBOT shows the potential in groups of elderly patients with severe LRC. The positive therapeutic outcome achieved in this 95-year-old patient, expands hyperbaric oxygen pressure options for elderly patients. Moreover, it also records a comprehensive dataset, including the time of the patient’s recurrence of hematuria and the subsequent trend of hematuria control, which contributes valuable evidence to the research on HBOT for the repair of bladder mucosal tissue.

## Data Availability

The original contributions presented in the study are included in the article/**Supplementary Material**. Further inquiries can be directed to the corresponding author.
