# Supplementary material for: Case report: Successful treatment of hyperbaric oxygen for radiation-induced hemorrhagic cystitis in a 95-year-old patient with bladder cancer
Source: Front Oncol. 2025 Feb 3;15:1410148. doi: 10.3389/fonc.2025.1410148 (PMC11830598; doi:10.3389/fonc.2025.1410148)
Supplement: Supplementary file 1 [file DataSheet1.docx]

Supplementary Material

Case report: Successful Treatment of Hyperbaric Oxygen for Radiation-Induced Hemorrhagic Cystitis in a 95-year-old with Bladder Cancer

**Li Lin^1,2^, Man He^2^, Yanyan Zeng^2^, Xiaoxiao Ni^2^, Yequn Guo^2^, Xiaojuan Xie^2^, Lingling Sun^2^ and Huai Huang^2*^**

*** Correspondence:** Huai Huang: [huanghuai1999@163.com](mailto:huanghuai1999@163.com)

## Supplementary Figures

**
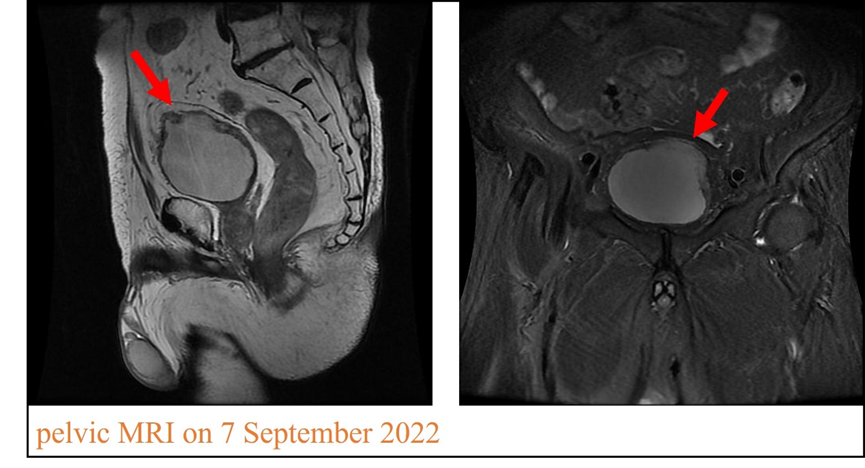
**

**Supplementary Figure S1.** The last pelvic MRI before initiating HBOT on 7 September 2022.

## Supplementary tables

**Table S1. Timeline of patient’s blood transfusion.**

| No | Date |
| --- | --- |
| 1 | 2022/6/16 |
| 2 | 2022/6/22 |
| 3 | 2022/7/2 |
| 4 | 2022/7/26 |
| 5 | 2022/7/28 |
| 6 | 2022/8/5 |
| 7 | 2022/8/12 |
| 8 | 2022/9/16 |
| 9 | 2022/9/27 |
| 10 | 2022/9/30 |
| 11 | 2022/10/6 |
| 12 | 2022/10/11 |
| 13 | 2022/10/17 |
| 14 | 2022/10/21 |
| 15 | 2022/10/23 |
| 16 | 2022/10/31 |
| 17 | 2022/11/1 |
| 18 | 2022/11/2 |
| 19 | 2022/11/7 |
| 20 | 2022/11/8 |
| 21 | 2022/11/9 |
| 22 | 2022/11/14 |
| 23 | 2022/11/15 |
| 24 | 2022/11/16 |
| 25 | 2022/11/20 |
| 26 | 2022/11/21 |
| 27 | 2022/11/22 |
| 28 | 2022/11/28 |
| 29 | 2022/11/29 |
| 30 | 2022/11/30 |
| 31 | 2022/12/15 |
| 32 | 2022/12/23 |
| 33 | 2022/12/24 |
| 34 | 2023/1/12 |
| 35 | 2023/1/13 |
| 36 | 2023/2/21 |

**Table S2. Timeline of patient’s HBOT.**

| Session | Date_HBOT | Time_HBOT | Oxygen inhalation duration each (min) | Number of replications | Pressure (ATA) |
| --- | --- | --- | --- | --- | --- |
| 1 | 2022/10/11 | 8:55 | 20 | 2 | 1.4 |
| 2 | 2022/10/12 | 8:45 | 20 | 2 | 1.4 |
| 3 | 2022/10/13 | 8:40 | 20 | 2 | 1.4 |
| 4 | 2022/10/14 | 8:00 | 35 | 2 | 1.5 |
| 5 | 2022/10/17 | 8:35 | 35 | 2 | 1.5 |
| 6 | 2022/10/18 | 8:30 | 35 | 2 | 1.5 |
| 7 | 2022/10/20 | 8:40 | 35 | 2 | 1.5 |
| 8 | 2022/10/21 | 8:30 | 35 | 2 | 1.5 |
| 9 | 2022/10/24 | 8:50 | 35 | 2 | 1.5 |
| 10 | 2022/10/25 | 8:45 | 35 | 2 | 1.5 |
| 11 | 2022/10/26 | 8:35 | 35 | 2 | 1.5 |
| 12 | 2022/10/27 | 8:40 | 35 | 2 | 1.5 |
| 13 | 2022/10/28 | 8:35 | 35 | 2 | 1.5 |
| 14 | 2022/10/31 | 8:40 | 35 | 2 | 1.5 |
| 15 | 2022/11/1 | 8:40 | 35 | 2 | 1.5 |
| 16 | 2022/11/2 | 8:40 | 35 | 2 | 1.5 |
| 17 | 2022/11/3 | 8:40 | 35 | 2 | 1.5 |
| 18 | 2022/11/4 | 8:40 | 35 | 2 | 1.5 |
| 19 | 2022/11/29 | 9:30 | 35 | 2 | 1.5 |
| 20 | 2022/11/30 | 9:30 | 35 | 2 | 1.5 |
| 21 | 2022/12/1 | 9:00 | 35 | 2 | 1.6 |
| 22 | 2022/12/2 | 9:00 | 35 | 2 | 1.6 |
| 23 | 2022/12/4 | 9:00 | 35 | 2 | 1.6 |
| 24 | 2022/12/5 | 9:00 | 35 | 2 | 1.6 |
| 25 | 2022/12/6 | 9:00 | 35 | 2 | 1.6 |
| 26 | 2022/12/7 | 9:00 | 35 | 2 | 1.6 |
| 27 | 2022/12/8 | 9:00 | 35 | 2 | 1.6 |
| 28 | 2022/12/9 | 9:00 | 35 | 2 | 1.6 |
| 29 | 2023/1/10 | 9:00 | 35 | 2 | 1.6 |
| 30 | 2023/1/11 | 9:00 | 35 | 2 | 1.6 |
| 31 | 2023/1/12 | 9:00 | 35 | 2 | 1.6 |
| 32 | 2023/1/13 | 9:00 | 35 | 2 | 1.6 |
| 33 | 2023/1/16 | 9:00 | 35 | 2 | 1.6 |
| 34 | 2023/2/6 | 9:00 | 35 | 2 | 1.6 |
| 35 | 2023/2/7 | 9:00 | 35 | 2 | 1.6 |
| 36 | 2023/2/8 | 9:00 | 35 | 2 | 1.6 |
| 37 | 2023/2/9 | 9:00 | 35 | 2 | 1.6 |
| 38 | 2023/2/10 | 9:00 | 35 | 2 | 1.6 |
| 39 | 2023/2/13 | 9:00 | 35 | 2 | 1.6 |
| 40 | 2023/2/14 | 9:00 | 35 | 2 | 1.6 |
| 41 | 2023/3/2 | 9:00 | 35 | 2 | 1.6 |
| 42 | 2023/3/3 | 9:00 | 35 | 2 | 1.6 |
| 43 | 2023/3/6 | 8:40 | 35 | 2 | 1.6 |
| 44 | 2023/3/7 | 8:50 | 35 | 2 | 1.6 |
| 45 | 2023/3/8 | 8:50 | 35 | 2 | 1.6 |
| 46 | 2023/3/9 | 8:50 | 35 | 2 | 1.6 |
| 47 | 2023/3/10 | 8:50 | 35 | 2 | 1.6 |
| 48 | 2023/3/13 | 9:00 | 35 | 2 | 1.6 |
| 49 | 2023/3/14 | 9:00 | 35 | 2 | 1.6 |
| 50 | 2023/3/15 | 9:00 | 35 | 2 | 1.6 |
| 51 | 2023/3/16 | 9:00 | 35 | 2 | 1.6 |
| 52 | 2023/3/17 | 9:00 | 35 | 2 | 1.6 |
| 53 | 2023/3/20 | 9:00 | 35 | 2 | 1.6 |
| 54 | 2023/3/21 | 8:40 | 35 | 2 | 1.6 |
| 55 | 2023/3/22 | 9:00 | 35 | 2 | 1.6 |
| 56 | 2023/3/23 | 9:00 | 35 | 2 | 1.6 |
| 57 | 2023/3/24 | 9:00 | 35 | 2 | 1.6 |
| 58 | 2023/3/25 | 15:00 | 35 | 2 | 1.6 |
| 59 | 2023/3/26 | 15:00 | 35 | 2 | 1.6 |
| 60 | 2023/3/27 | 9:00 | 35 | 2 | 1.6 |
| 61 | 2023/3/28 | 9:00 | 35 | 2 | 1.6 |
| 62 | 2023/3/29 | 9:00 | 35 | 2 | 1.6 |
| 63 | 2023/3/30 | 9:00 | 35 | 2 | 1.6 |
| 64 | 2023/3/31 | 9:00 | 35 | 2 | 1.6 |
| 65 | 2023/4/1 | 14:50 | 35 | 2 | 1.6 |
| 66 | 2023/4/2 | 15:00 | 35 | 2 | 1.6 |
| 67 | 2023/4/3 | 8:40 | 35 | 2 | 1.6 |
| 68 | 2023/4/4 | 8:30 | 35 | 2 | 1.6 |
| 69 | 2023/4/5 | 15:00 | 35 | 2 | 1.6 |
| 70 | 2023/4/6 | 8:30 | 35 | 2 | 1.6 |
| 71 | 2023/4/7 | 8:30 | 35 | 2 | 1.6 |
| 72 | 2023/4/8 | 14:30 | 35 | 2 | 1.6 |
| 73 | 2023/4/9 | 14:30 | 35 | 2 | 1.6 |
| 74 | 2023/4/10 | 8:20 | 35 | 2 | 1.6 |
| 75 | 2023/4/11 | 8:20 | 35 | 2 | 1.6 |
| 76 | 2023/4/12 | 8:00 | 35 | 2 | 1.6 |
| 77 | 2023/4/13 | 8:00 | 35 | 2 | 1.6 |
| 78 | 2023/4/14 | 8:00 | 35 | 2 | 1.6 |
| 79 | 2023/4/18 | 8:20 | 35 | 2 | 1.6 |
| 80 | 2023/4/19 | 8:20 | 35 | 2 | 1.6 |
| 81 | 2023/4/20 | 8:20 | 35 | 2 | 1.6 |
| 82 | 2023/4/21 | 8:20 | 35 | 2 | 1.6 |
| 83 | 2023/4/22 | 15:00 | 35 | 2 | 1.6 |
| 84 | 2023/4/23 | 8:20 | 35 | 2 | 1.6 |
| 85 | 2023/4/24 | 8:20 | 35 | 2 | 1.6 |
| 86 | 2023/4/25 | 8:20 | 35 | 2 | 1.6 |
| 87 | 2023/4/26 | 8:30 | 35 | 2 | 1.6 |
| 88 | 2023/4/27 | 8:30 | 35 | 2 | 1.6 |
| 89 | 2023/4/28 | 8:30 | 35 | 2 | 1.6 |
| 90 | 2023/4/29 | 15:00 | 35 | 2 | 1.6 |
| 91 | 2023/5/3 | 15:00 | 35 | 2 | 1.6 |
| 92 | 2023/5/4 | 8:50 | 35 | 2 | 1.6 |
| 93 | 2023/5/5 | 8:50 | 35 | 2 | 1.6 |
| 94 | 2023/5/6 | 8:50 | 35 | 2 | 1.6 |
| 95 | 2023/5/7 | 15:00 | 35 | 2 | 1.6 |
| 96 | 2023/5/8 | 8:50 | 35 | 2 | 1.6 |
| 97 | 2023/5/9 | 8:50 | 35 | 2 | 1.6 |
| 98 | 2023/5/10 | 8:50 | 35 | 2 | 1.6 |
| 99 | 2023/5/11 | 8:50 | 35 | 2 | 1.6 |
| 100 | 2023/5/12 | 8:50 | 35 | 2 | 1.6 |
| 101 | 2023/5/13 | 15:00 | 35 | 2 | 1.6 |
| 102 | 2023/5/14 | 15:00 | 35 | 2 | 1.6 |
| 103 | 2023/5/15 | 8:45 | 35 | 2 | 1.6 |
| 104 | 2023/5/16 | 8:45 | 35 | 2 | 1.6 |
| 105 | 2023/5/17 | 8:45 | 35 | 2 | 1.6 |
| 106 | 2023/5/18 | 8:45 | 35 | 2 | 1.6 |
| 107 | 2023/5/19 | 8:00 | 35 | 2 | 1.6 |
| 108 | 2023/5/20 | 15:00 | 35 | 2 | 1.6 |
| 109 | 2023/5/21 | 15:00 | 35 | 2 | 1.6 |
| 110 | 2023/5/22 | 8:40 | 35 | 2 | 1.6 |
| 111 | 2023/5/23 | 8:40 | 35 | 2 | 1.6 |
| 112 | 2023/5/24 | 8:40 | 35 | 2 | 1.6 |
| 113 | 2023/5/25 | 8:40 | 35 | 2 | 1.6 |
| 114 | 2023/5/26 | 8:40 | 35 | 2 | 1.6 |
| 115 | 2023/5/27 | 15:00 | 35 | 2 | 1.6 |
| 116 | 2023/5/28 | 15:00 | 35 | 2 | 1.6 |
| 117 | 2023/5/29 | 8:45 | 35 | 2 | 1.6 |
| 118 | 2023/5/30 | 8:40 | 35 | 2 | 1.6 |
| 119 | 2023/5/31 | 8:40 | 35 | 2 | 1.6 |
| 120 | 2023/6/1 | 8:40 | 35 | 2 | 1.6 |
| 121 | 2023/6/7 | 8:35 | 35 | 2 | 1.6 |
| 122 | 2023/6/8 | 8:40 | 35 | 2 | 1.6 |
| 123 | 2023/6/9 | 8:40 | 35 | 2 | 1.6 |
| 124 | 2023/6/12 | 8:25 | 35 | 2 | 1.6 |
| 125 | 2023/6/13 | 8:20 | 35 | 2 | 1.6 |
| 126 | 2023/6/14 | 8:30 | 35 | 2 | 1.6 |
| 127 | 2023/6/15 | 8:20 | 35 | 2 | 1.6 |
| 128 | 2023/6/16 | 8:20 | 35 | 2 | 1.6 |
| 129 | 2023/6/19 | 8:30 | 35 | 2 | 1.6 |
| 130 | 2023/6/20 | 8:30 | 35 | 2 | 1.6 |
| 131 | 2023/6/21 | 8:30 | 35 | 2 | 1.6 |
| 132 | 2023/6/25 | 8:30 | 35 | 2 | 1.6 |
| 133 | 2023/6/26 | 9:00 | 35 | 2 | 1.6 |
| 134 | 2023/6/27 | 8:25 | 35 | 2 | 1.6 |
| 135 | 2023/6/28 | 8:25 | 35 | 2 | 1.6 |
| 136 | 2023/6/29 | 9:00 | 35 | 2 | 1.6 |
| 137 | 2023/6/30 | 8:50 | 35 | 2 | 1.6 |
| 138 | 2023/7/3 | 8:50 | 35 | 2 | 1.6 |
| 139 | 2023/7/4 | 8:50 | 35 | 2 | 1.6 |
| 140 | 2023/7/5 | 8:50 | 35 | 2 | 1.6 |
| 141 | 2023/7/6 | 8:50 | 35 | 2 | 1.6 |
| 142 | 2023/7/7 | 8:50 | 35 | 2 | 1.6 |
| 143 | 2023/7/10 | 8:50 | 35 | 2 | 1.6 |
| 144 | 2023/7/11 | 8:40 | 35 | 2 | 1.6 |
| 145 | 2023/7/12 | 8:40 | 35 | 2 | 1.6 |
| 146 | 2023/7/13 | 8:40 | 35 | 2 | 1.6 |
| 147 | 2023/7/14 | 8:40 | 35 | 2 | 1.6 |
| 148 | 2023/7/17 | 8:40 | 35 | 2 | 1.6 |
| 149 | 2023/7/18 | 8:40 | 35 | 2 | 1.6 |
| 150 | 2023/7/19 | 8:00 | 35 | 2 | 1.6 |
| 151 | 2023/7/31 | 8:40 | 35 | 2 | 1.6 |
| 152 | 2023/8/1 | 8:40 | 35 | 2 | 1.6 |
| 153 | 2023/8/2 | 8:40 | 35 | 2 | 1.6 |
| 154 | 2023/8/3 | 8:40 | 35 | 2 | 1.6 |
| 155 | 2023/8/4 | 8:40 | 35 | 2 | 1.6 |
| 156 | 2023/8/7 | 8:40 | 35 | 2 | 1.6 |
| 157 | 2023/8/8 | 8:40 | 35 | 2 | 1.6 |
| 158 | 2023/8/9 | 8:40 | 35 | 2 | 1.6 |
| 159 | 2023/8/10 | 8:40 | 35 | 2 | 1.6 |
| 160 | 2023/8/11 | 8:40 | 35 | 2 | 1.6 |
| 161 | 2023/8/14 | 8:40 | 35 | 2 | 1.6 |
| 162 | 2023/8/15 | 8:50 | 35 | 2 | 1.6 |
| 163 | 2023/8/21 | 8:50 | 35 | 2 | 1.6 |
| 164 | 2023/8/22 | 9:00 | 35 | 2 | 1.6 |
| 165 | 2023/8/23 | 8:50 | 35 | 2 | 1.6 |
| 166 | 2023/8/24 | 8:45 | 35 | 2 | 1.6 |
| 167 | 2023/8/25 | 8:40 | 35 | 2 | 1.6 |
| 168 | 2023/8/28 | 8:40 | 35 | 2 | 1.6 |
| 169 | 2023/8/29 | 8:40 | 35 | 2 | 1.6 |
| 170 | 2023/8/30 | 8:40 | 35 | 2 | 1.6 |
| 171 | 2023/8/31 | 8:40 | 35 | 2 | 1.6 |
| 172 | 2023/9/1 | 8:40 | 35 | 2 | 1.6 |
| 173 | 2023/9/4 | 8:50 | 35 | 2 | 1.6 |
| 174 | 2023/9/5 | 8:45 | 35 | 2 | 1.6 |
| 175 | 2023/9/6 | 8:45 | 35 | 2 | 1.6 |
| 176 | 2023/9/7 | 8:45 | 35 | 2 | 1.6 |
| 177 | 2023/9/8 | 8:55 | 35 | 2 | 1.6 |
| 178 | 2023/9/11 | 8:40 | 35 | 2 | 1.6 |
| 179 | 2023/9/12 | 8:40 | 35 | 2 | 1.6 |
| 180 | 2023/9/13 | 8:40 | 35 | 2 | 1.6 |
| 181 | 2023/9/14 | 8:40 | 35 | 2 | 1.6 |
| 182 | 2023/9/15 | 8:40 | 35 | 2 | 1.6 |
| 183 | 2023/9/18 | 8:40 | 35 | 2 | 1.6 |
| 184 | 2023/9/19 | 8:00 | 35 | 2 | 1.6 |
| 185 | 2023/9/20 | 8:00 | 35 | 2 | 1.6 |
| 186 | 2023/10/9 | 8:50 | 35 | 2 | 1.6 |
| 187 | 2023/10/10 | 9:10 | 35 | 2 | 1.6 |
| 188 | 2023/10/11 | 8:55 | 35 | 2 | 1.6 |
| 189 | 2023/10/12 | 9:00 | 35 | 2 | 1.6 |
| 190 | 2023/10/13 | 9:10 | 35 | 2 | 1.6 |
| 191 | 2023/10/16 | 9:00 | 35 | 2 | 1.6 |
| 192 | 2023/10/17 | 9:00 | 35 | 2 | 1.6 |
| 193 | 2023/10/18 | 9:00 | 35 | 2 | 1.6 |
| 194 | 2023/10/19 | 9:00 | 35 | 2 | 1.6 |
| 195 | 2023/10/20 | 9:00 | 35 | 2 | 1.6 |
| 196 | 2023/10/23 | 9:00 | 35 | 2 | 1.6 |

**Table S3. Timeline of patient’s urine RBC and RBC-M count.**

| Date of routine urinalysis | RBC (RBC/μL) | RBC-M (RBC/HPF) |
| --- | --- | --- |
| 2021/2/23 | 7838.7 | 1411 |
| 2021/3/3 | 3489.9 | 628.2 |
| 2021/3/31 | 19.4 | 3.5 |
| 2021/4/13 | 2642.8 | 475.7 |
| 2021/4/16 | 503.1 | 90.6 |
| 2021/4/17 | 612.8 | 110.3 |
| 2021/4/19 | 837.7 | 150.8 |
| 2021/4/19 | 755.8 | 136 |
| 2021/4/20 | 439 | 79 |
| 2021/5/11 | 1831.3 | 329.6 |
| 2021/5/14 | 7.7 | 1.4 |
| 2021/5/26 | 23.2 | 4.2 |
| 2021/5/26 | 22 | 4 |
| 2021/5/31 | 403.4 | 72.6 |
| 2021/6/1 | 1631.3 | 293.6 |
| 2021/6/4 | 7.4 | 1.3 |
| 2021/6/9 | 35.9 | 6.5 |
| 2021/6/11 | 106.1 | 19.1 |
| 2021/6/11 | 93.7 | 16.9 |
| 2021/6/14 | 666.6 | 120 |
| 2021/6/17 | 25 | 4.5 |
| 2021/6/19 | 523.2 | 94.2 |
| 2021/6/23 | 196.1 | 35.3 |
| 2021/6/25 | 20 | 3.6 |
| 2021/6/28 | 463 | 83.3 |
| 2021/6/30 | 2099 | 377.8 |
| 2021/7/1 | 1808.9 | 325.6 |
| 2021/7/2 | 357.4 | 64.3 |
| 2021/7/3 | 19.2 | 3.5 |
| 2021/7/5 | 42.1 | 7.6 |
| 2021/7/6 | 31.6 | 5.7 |
| 2021/7/8 | 29.4 | 5.3 |
| 2022/3/8 | 22985.1 | 4137.3 |
| 2022/3/29 | 6515.8 | 1172.8 |
| 2022/6/16 | 13030.1 | 2345.4 |
| 2022/6/23 | 3412.8 | 614.3 |
| 2022/6/24 | 30226.6 | 5440.8 |
| 2022/6/25 | 61.9 | 11.1 |
| 2022/6/26 | 68.3 | 12.3 |
| 2022/6/27 | 14855.6 | 2674 |
| 2022/6/28 | 217.3 | 39.1 |
| 2022/6/28 | 83969.3 | 15114.5 |
| 2022/6/29 | 29532 | 5315.8 |
| 2022/7/1 | 33102.4 | 5958.4 |
| 2022/7/26 | 91 | 16.4 |
| 2022/7/27 | 78252.8 | 14085.5 |
| 2022/7/29 | 5209.6 | 937.7 |
| 2022/7/30 | 27085.6 | 4875.4 |
| 2022/7/31 | 21563.8 | 3881.5 |
| 2022/7/31 | 6366.4 | 1146 |
| 2022/8/1 | 205.5 | 37 |
| 2022/8/1 | 3.2 | 0.6 |
| 2022/8/2 | 7234.8 | 1302.3 |
| 2022/8/4 | 2495.6 | 449.2 |
| 2022/8/4 | 5018 | 903.2 |
| 2022/8/5 | 9242.3 | 1663.6 |
| 2022/8/8 | 57.7 | 10.4 |
| 2022/8/9 | 32.6 | 5.9 |
| 2022/8/10 | 6 | 1.1 |
| 2022/8/12 | 12.2 | 2.2 |
| 2022/9/2 | 56 | 10.1 |
| 2022/9/5 | 33341.6 | 6001.5 |
| 2022/9/6 | 667.5 | 120.2 |
| 2022/9/7 | 8490.6 | 1528.3 |
| 2022/9/8 | 18790.1 | 3382.2 |
| 2022/9/9 | 1202 | 216.4 |
| 2022/9/9 | 8900.2 | 1602 |
| 2022/9/9 | 9350 | 1683 |
| 2022/9/11 | 2499.9 | 450 |
| 2022/9/13 | 3259.1 | 586.6 |
| 2022/9/15 | 3589 | 664 |
| 2022/9/16 | 70208 | 12637.5 |
| 2022/9/17 | 93187.6 | 16773.8 |
| 2022/9/18 | 94763.2 | 17057.4 |
| 2022/9/20 | 95621 | 17211.8 |
| 2022/9/22 | 74394.5 | 13391 |
| 2022/9/26 | 18931 | 3404.3 |
| 2022/10/2 | 95041.7 | 17107.5 |
| 2022/10/3 | 99999 | 17999.8 |
| 2022/10/15 | 80318.2 | 14457.3 |
| 2022/10/18 | 61264.8 | 11027.7 |
| 2022/10/21 | 122550 | 22059 |
| 2022/10/22 | 66097.2 | 11897.5 |
| 2022/10/23 | 64414.5 | 11594.6 |
| 2022/10/24 | 63124.3 | 11362.4 |
| 2022/10/25 | 65214.2 | 11738.6 |
| 2022/10/27 | 63210.1 | 11377.8 |
| 2022/10/28 | 85044.9 | 15308.1 |
| 2022/10/29 | 23924.9 | 4306.5 |
| 2022/10/31 | 30552 | 5499.4 |
| 2022/11/3 | 3441.3 | 619.4 |
| 2022/11/4 | 3216.9 | 579 |
| 2022/11/5 | 29659.1 | 5338.6 |
| 2022/11/6 | 210859 | 37954.6 |
| 2022/11/7 | 30894.2 | 5561 |
| 2022/11/8 | 13654.9 | 2457.9 |
| 2022/11/9 | 46325.1 | 8338.5 |
| 2022/11/10 | 95154.4 | 17127.8 |
| 2022/11/11 | 1580.3 | 284.5 |
| 2022/11/12 | 96712.8 | 17408.3 |
| 2022/11/13 | 146234.4 | 26322.2 |
| 2022/11/14 | 11273.3 | 2029.2 |
| 2022/11/15 | 69949.3 | 12590.9 |
| 2022/11/16 | 56819.5 | 10227.5 |
| 2022/11/17 | 58567 | 10542.1 |
| 2022/11/18 | 56892 | 10240.6 |
| 2022/11/19 | 79999.6 | 14399.6 |
| 2022/11/20 | 180.8 | 34.2 |
| 2022/11/21 | 65210.1 | 11737.8 |
| 2022/11/22 | 50710 | 9127.8 |
| 2022/11/23 | 9566.9 | 1722 |
| 2022/11/24 | 1214.7 | 218.6 |
| 2022/11/25 | 8394.4 | 1511 |
| 2022/11/26 | 14804.3 | 2664.8 |
| 2022/11/27 | 65572.3 | 11803 |
| 2022/11/28 | 51315.5 | 9236.8 |
| 2022/11/29 | 53642.3 | 9655.6 |
| 2022/11/30 | 5165.8 | 929.8 |
| 2022/12/1 | 4493.1 | 808.8 |
| 2022/12/2 | 5040.9 | 907.4 |
| 2022/12/3 | 3151.3 | 567.2 |
| 2022/12/4 | 5029.6 | 905.3 |
| 2022/12/4 | 2965.9 | 533.9 |
| 2022/12/5 | 24412.8 | 4394.3 |
| 2022/12/6 | 21356.3 | 3844.1 |
| 2022/12/6 | 22230.2 | 4001.4 |
| 2022/12/7 | 30832.8 | 5549.9 |
| 2022/12/8 | 24659.7 | 4438.7 |
| 2022/12/9 | 57804.6 | 10404.8 |
| 2022/12/10 | 4975.8 | 895.6 |
| 2022/12/11 | 30336.6 | 5460.6 |
| 2022/12/12 | 23986.8 | 4317.6 |
| 2022/12/13 | 7333 | 1319.9 |
| 2022/12/14 | 6568.4 | 1182.3 |
| 2022/12/15 | 12365 | 2225.7 |
| 2022/12/16 | 21205.3 | 3817 |
| 2022/12/17 | 26341.4 | 4741.4 |
| 2022/12/18 | 23652 | 4257.4 |
| 2022/12/19 | 19090.4 | 3436.3 |
| 2022/12/20 | 18365.3 | 3305.8 |
| 2022/12/21 | 10558 | 1900.4 |
| 2022/12/22 | 4038.9 | 727 |
| 2022/12/23 | 22680.5 | 4082.5 |
| 2022/12/24 | 7501 | 1350.2 |
| 2022/12/25 | 36537.8 | 6576.8 |
| 2022/12/26 | 16415.9 | 2954.9 |
| 2022/12/27 | 38015.8 | 6842.8 |
| 2022/12/28 | 34734.5 | 6252.2 |
| 2022/12/29 | 60658 | 10918.4 |
| 2022/12/30 | 39622.1 | 7132 |
| 2022/12/31 | 72814.7 | 13106.6 |
| 2023/1/2 | 18204 | 3276.7 |
| 2023/1/4 | 23754.2 | 4275.8 |
| 2023/1/5 | 20400.9 | 3672.2 |
| 2023/1/6 | 46144.3 | 8306 |
| 2023/1/7 | 28173.8 | 5071.3 |
| 2023/1/8 | 13374.3 | 2407.4 |
| 2023/1/9 | 30861.6 | 5555.1 |
| 2023/1/10 | 18221 | 3279.8 |
| 2023/1/11 | 8976.4 | 1615.8 |
| 2023/1/12 | 4923.9 | 886.3 |
| 2023/1/13 | 67793.7 | 12202.9 |
| 2023/1/14 | 17756.6 | 3196.2 |
| 2023/1/15 | 3540.3 | 637.3 |
| 2023/1/16 | 163 | 29.3 |
| 2023/1/17 | 2130.7 | 383.5 |
| 2023/1/18 | 889.6 | 160.1 |
| 2023/1/19 | 4130.7 | 743.5 |
| 2023/1/20 | 597.5 | 107.6 |
| 2023/1/21 | 2091.2 | 376.4 |
| 2023/1/22 | 3596.1 | 647.3 |
| 2023/1/23 | 4210 | 757.8 |
| 2023/1/24 | 9184 | 1653.1 |
| 2023/1/25 | 3930.7 | 707.5 |
| 2023/1/26 | 10213.2 | 1838.4 |
| 2023/1/27 | 1691.8 | 304.5 |
| 2023/1/28 | 1734.2 | 312.2 |
| 2023/1/29 | 1996 | 359.3 |
| 2023/1/30 | 1652.7 | 297.5 |
| 2023/1/31 | 2347.7 | 422.6 |
| 2023/2/1 | 827.3 | 148.9 |
| 2023/2/2 | 995.9 | 179.3 |
| 2023/2/3 | 1900.4 | 342.1 |
| 2023/2/4 | 787.2 | 141.7 |
| 2023/2/5 | 647.8 | 116.6 |
| 2023/2/6 | 15091.4 | 2716.5 |
| 2023/2/7 | 12530.2 | 2255.4 |
| 2023/2/8 | 13683 | 2462.9 |
| 2023/2/9 | 8168.1 | 1470.3 |
| 2023/2/10 | 4733.9 | 852.1 |
| 2023/2/11 | 12988.6 | 2337.9 |
| 2023/2/12 | 5782.5 | 1040.9 |
| 2023/2/13 | 9769.7 | 1758.5 |
| 2023/2/14 | 7904.3 | 1422.8 |
| 2023/2/15 | 20745.5 | 3734.2 |
| 2023/2/15 | 8919.3 | 1605.5 |
| 2023/2/16 | 41794.6 | 7523 |
| 2023/2/17 | 6706.4 | 1207.2 |
| 2023/2/18 | 9711.5 | 1748.1 |
| 2023/2/20 | 10703.6 | 1926.6 |
| 2023/2/21 | 40891.2 | 7360.4 |
| 2023/2/22 | 27076.2 | 4873.7 |
| 2023/2/23 | 7617 | 1371.1 |
| 2023/2/24 | 6823 | 1228.1 |
| 2023/2/25 | 8576.6 | 1543.8 |
| 2023/2/26 | 6877.5 | 1238 |
| 2023/2/27 | 27268.8 | 4908.4 |
| 2023/2/28 | 3790.3 | 682.3 |
| 2023/3/1 | 5419.9 | 975.6 |
| 2023/3/2 | 6096.8 | 1097.4 |
| 2023/3/3 | 10651.4 | 1917.3 |
| 2023/3/4 | 1880.1 | 338.4 |
| 2023/3/5 | 8970.2 | 1614.6 |
| 2023/3/6 | 16171.7 | 2910.9 |
| 2023/3/7 | 19900.6 | 3582.1 |
| 2023/3/8 | 17244.9 | 3104.1 |
| 2023/3/9 | 52558.2 | 9460.5 |
| 2023/3/10 | 43895.9 | 7901.3 |
| 2023/3/11 | 47558 | 8560.4 |
| 2023/3/12 | 89319.9 | 16077.6 |
| 2023/3/13 | 56899 | 10241.8 |
| 2023/3/14 | 45096.5 | 8117.4 |
| 2023/3/15 | 51234 | 9222.1 |
| 2023/3/16 | 19064.5 | 3431.6 |
| 2023/3/17 | 17809.5 | 3205.7 |
| 2023/3/18 | 19353.7 | 3483.7 |
| 2023/3/19 | 42914.9 | 7724.7 |
| 2023/3/20 | 32698 | 5885.6 |
| 2023/3/21 | 12564 | 2261.5 |
| 2023/3/22 | 72978.8 | 13136.2 |
| 2023/3/23 | 52562 | 9461.2 |
| 2023/3/24 | 17762.5 | 3197.3 |
| 2023/3/25 | 48601.3 | 8748.2 |
| 2023/3/27 | 28587.3 | 5145.7 |
| 2023/3/28 | 12690 | 2284.2 |
| 2023/3/29 | 10521.7 | 1893.9 |
| 2023/3/30 | 2918.7 | 525.4 |
| 2023/3/31 | 5539.5 | 997.1 |
| 2023/4/1 | 7449.5 | 1340.9 |
| 2023/4/2 | 9682.5 | 1742.9 |
| 2023/4/3 | 882.8 | 158.9 |
| 2023/4/4 | 2347.2 | 422.5 |
| 2023/4/5 | 47.5 | 8.6 |
| 2023/4/6 | 92.4 | 16.6 |
| 2023/4/7 | 561.8 | 101.1 |
| 2023/4/8 | 137.6 | 24.8 |
| 2023/4/9 | 73.8 | 13.3 |
| 2023/4/10 | 47.3 | 8.5 |
| 2023/4/11 | 307.7 | 55.4 |
| 2023/4/12 | 172.4 | 31 |
| 2023/4/13 | 279.7 | 50.3 |
| 2023/4/14 | 869.2 | 156.5 |
| 2023/4/16 | 207.6 | 37.4 |
| 2023/4/17 | 58.7 | 10.6 |
| 2023/4/18 | 32.9 | 5.9 |
| 2023/4/19 | 13.8 | 2.5 |
| 2023/4/20 | 510.3 | 91.9 |
| 2023/4/21 | 77.2 | 13.9 |
| 2023/4/22 | 81.3 | 14.6 |
| 2023/4/25 | 38.6 | 6.9 |
| 2023/4/27 | 154.2 | 27.8 |
| 2023/4/29 | 17.5 | 3.2 |
| 2023/5/1 | 51.8 | 9.3 |
| 2023/5/3 | 86.5 | 15.6 |
| 2023/5/8 | 91.8 | 16.5 |
| 2023/5/9 | 104.8 | 18.9 |
| 2023/5/11 | 34.4 | 6.2 |
| 2023/5/13 | 99.5 | 17.9 |
| 2023/5/16 | 6.2 | 1.1 |
| 2023/5/18 | 16.1 | 2.9 |
| 2023/5/22 | 31.5 | 5.7 |
| 2023/5/24 | 28.1 | 5.1 |
| 2023/5/26 | 78.8 | 14.2 |
| 2023/5/29 | 17.7 | 3.2 |
| 2023/5/31 | 5 | 0.9 |
| 2023/10/26 | 4.4 | 0.8 |

**Table S4. Timeline of patient’s HGB level.**

| Date of routine bloodwork | HGB (g/L) |
| --- | --- |
| 2020/12/4 | 109 |
| 2021/3/2 | 99 |
| 2021/3/31 | 101 |
| 2021/4/13 | 105 |
| 2021/5/11 | 98 |
| 2021/5/25 | 102 |
| 2021/6/3 | 102 |
| 2021/7/6 | 103 |
| 2022/6/16 | 56 |
| 2022/6/21 | 65 |
| 2022/6/29 | 64 |
| 2022/7/1 | 73 |
| 2022/7/26 | 52 |
| 2022/7/28 | 61 |
| 2022/7/30 | 78 |
| 2022/8/2 | 74 |
| 2022/8/5 | 68 |
| 2022/8/9 | 81 |
| 2022/8/12 | 78 |
| 2022/9/2 | 104 |
| 2022/9/6 | 99 |
| 2022/9/9 | 101 |
| 2022/9/13 | 89 |
| 2022/9/16 | 76 |
| 2022/9/20 | 85 |
| 2022/9/27 | 60 |
| 2022/9/30 | 72 |
| 2022/10/5 | 76 |
| 2022/10/11 | 73 |
| 2022/10/22 | 77 |
| 2022/10/31 | 47 |
| 2022/11/7 | 61 |
| 2022/11/9 | 73 |
| 2022/11/14 | 60 |
| 2022/11/20 | 60 |
| 2022/11/28 | 53 |
| 2022/11/30 | 83 |
| 2022/12/4 | 107 |
| 2022/12/9 | 89 |
| 2022/12/13 | 93 |
| 2022/12/16 | 89 |
| 2022/12/21 | 78 |
| 2022/12/26 | 102 |
| 2022/12/28 | 96 |
| 2022/12/30 | 90 |
| 2023/1/3 | 82 |
| 2023/1/6 | 80 |
| 2023/1/9 | 79 |
| 2023/1/12 | 76 |
| 2023/1/15 | 96 |
| 2023/1/16 | 104 |
| 2023/1/17 | 84 |
| 2023/1/18 | 94 |
| 2023/1/20 | 102 |
| 2023/1/23 | 90 |
| 2023/1/25 | 83 |
| 2023/1/27 | 91 |
| 2023/1/29 | 87 |
| 2023/1/31 | 90 |
| 2023/2/7 | 101 |
| 2023/2/14 | 96 |
| 2023/2/19 | 87 |
| 2023/2/21 | 76 |
| 2023/2/25 | 106 |
| 2023/3/10 | 101 |
| 2023/3/18 | 87 |
| 2023/3/28 | 86 |
| 2023/4/22 | 85 |
| 2023/6/1 | 106 |
| 2023/10/26 | 106 |
